# Role of plasma matrix-metalloproteases (MMPs) and their polymorphisms (SNPs) in sepsis development and outcome in ICU patients

**DOI:** 10.1038/srep05002

**Published:** 2014-05-16

**Authors:** Guadalupe Martin, Víctor Asensi, A. Hugo Montes, Julio Collazos, Victoria Alvarez, José A. Carton, Francisco Taboada, Eulalia Valle-Garay

**Affiliations:** 1 Critical Care, Hospital Universitario Central de Asturias (HUCA); 2 Infectious Diseases, Hospital Universitario Central de Asturias (HUCA); 3 Biochemistry and Molecular Biology, Oviedo University School of Medicine, Oviedo; 4 Infectious Diseases, Hospital de Galdacano, Vizcaya, all in Spain; 5 Molecular Genetics Unit-Nephrology Research Institute, Hospital Universitario Central de Asturias (HUCA)

## Abstract

Matrix-metalloproteases (MMPs) and their tissue-inhibitors (TIMPs), modulated by different single nucleotide polymorphisms (SNPs), are critical in sepsis development. Ninety ICU severely septic and 91 ICU uninfected patients were prospectively studied. *MMP-1 (−1607 1G/2G)*, *MMP-3 (−1612 5A/6A)*, *MMP-8 (−799 C/T)*, *MMP-9 (−1562 C/T)*, and *MMP-1*3 (*−77A/G*) SNPs were genotyped. Plasma MMPs (-1, -2, -3, -8, -9, -10, -13) and TIMPs (-1,-2,-4) were measured. *AA* homozygotes and *A* allele carriers of *MMP-13* (*−77 A/G*) and *1G2G* carriers of the *MMP-1 (−1607 1G/2G)* SNPs frequencies were different between septic and uninfected patients (p < 0.05), as well as plasma MMP-3, -8, -9 -10 and TIMP-2 levels (p < 0.04). No differences in MMPs levels among *MMP-13* or *MMP-1* SNPs genotypes carriers were observed. The area under the ROC curve for MMP-8 in the diagnosis of sepsis was 0.87 (95% CI 0.82–0.92), and that of CRP was 0.98 (0.94–0.998), whereas the area of MMP-9 in the detection of non-septic state was 0.73 (0.65–0.80), p < 0.0001 for all curves. Sepsis associated with increased MMP-8 and decreased MMP-9 levels in multivariate analysis (p < 0.0002). We report for the first time an association between *MMP-13* and *MMP-1* SNPs and sepsis. An independent association of MMP-8 and MMP-9 levels with sepsis was also observed.

Septic shock is the most common cause of death in the Intensive Care Units (ICU). Despite modern intensive care and antibiotic treatments the mortality of sepsis still remains high, ranging from 20% to 30% in septic shock^1^,^2^. The role of the immune system in the pathophysiology of septic shock (cytokines, neutrophils, monocytes, macrophages) has been well documented but little is known regarding the role of extracellular matrix metalloproteases (MMPs)^3^,^4^.

MMPS are a family of zinc-dependent endoproteases that share amino-acid sequences, structural domains and substrates and can degrade the extracellular matrix (ECM) proteins. Their activity depends on activation of MMPs zymogens and is influenced by tissue inhibitors (TIMPs). MMPs are involved in the response to tissue injury and inflammation and are stimulated by cytokines. MMPs activity is increased in inflammatory diseases, acute respiratory distress syndrome (ARDS) and in response to endotoxin injection. Some MMPs, such as MMP-8 and -9, are stored in neutrophils granules and are liberated by endotoxin. MMPs release membrane-bound cytokines such as TNF-α. MMPs cleavage of ECM collagen present in membranes basement might help neutrophils crossing blood and lymph circulation into sites of infection^5^,^6^. There is a large and ever increasing family of mammalian MMPs that are broadly divided in different subfamilies according to their substrate specificity. Gelatinases (MMP-2 and -9) have been related to sepsis so far although less attention has been paid to collagenases (MMP-1, - 13) and stromelysins (MMP-3, -10). This is important considering the central role played by MMP-3 and especially MMP-13 in the MMPs activation cascade^7^. Previous studies have found increased serum levels of most of the MMPs and TIMPs in sepsis although results are far from clear^8^,^9^,^10^,^11^,^12^,^13^,^14^,^15^. Only three studies centered on the time course of MMPs and TIMPs so far, two of them only tangentially^12^,^13^,^14^. Different polymorphisms (SNPs) of *MMPs* and *TIMPs* have been described. Some of them such as the *MMP-3 (-1612 5A/6A)*, *MMP-9(-1562 C*/T) and *MMP-13 (-77 A/G)* are located in the *MMPs* genes promoter region and induce changes in *MMPs* genes mRNA and protein expression^16^. These functional *MMPs* SNPs are associated mostly with cardiovascular disease susceptibility, but also with cancer, rheumatic diseases and other conditions, such as endometriosis^16^,^17^,^18^,^19^,^20^,^21^,^22^,^23^,^24^. To our knowledge, only two papers on MMPs SNPs and infection have been published, one reporting an association of a *MMP9* SNP with periodontitis susceptibility^25^ and a second, from our group, of a *MMP1* SNP with bacterial osteomyelitis^26^. Very recently an association between a *TIMP1* SNP and sepsis mortality has been published^27^.

The aims of this study were: 1. to investigate the baseline and time course plasma levels of MMPs and TIMPs in ICU septic and uninfected patients; 2. to investigate whether *MMPs* SNPs might associate with susceptibility to sepsis or influence the sepsis outcome, with/out associated changes in plasma MMPs and TIMPs levels. In order to answer these questions, plasma levels of different MMPs and TIMPs were measured and different SNPs of *MMPs* were genotyped in septic and uninfected control patients. The time course of MMPs and TIMPs at days 1, 3 and 7 of ICU admission in a subgroup of septic patients and uninfected controls was also analyzed.

## Methods

### Patients

Ninety ICU patients with severe sepsis admitted to the ICU of the Hospital Universitario Central de Asturias (HUCA) in Oviedo, Spain, between May 2007 and August 2010 were enrolled in the study. Patients were enrolled as septic if fulfilled the diagnosis of severe sepsis according to the 1992 International Sepsis Definitions Conference Criteria modified in 2003^28^,^29^. In addition, all septic patients had positive blood cultures or a microbiologically-demonstrated source of bacterial infection at ICU admission. All infections were community-acquired. Patients with cancer, HIV infection, transplantation or other causes of immunodepression were excluded. Ninety one uninfected patients admitted to the ICU for other non-infective diseases, mostly severe trauma and brain strokes, were used as controls. If a control patient developed an ICU infection during the follow-up was excluded from the control group. APACHE II scores and number of organ failures were calculated in septic patients and uninfected controls. Patients and controls were members of a homogeneous Caucasian population, and were residents of the same region (Asturias, Northern Spain) that has a small foreign immigrant population (less than 5%). Each participant or their legal representatives gave informed consent for the study, which was approved by the Ethics Committee of the HUCA. Organ dysfunction failures were defined according to Marshall^30^. Treatment of organ failures, volume resuscitation, and supportive therapy for sepsis were based on currently applied guidelines^31^. Fluid resuscitation with crystalloids and colloids, early broad spectrum antibiotic treatment (most commonly broad spectrum β-lactamics such as carbapenems or piperacillin-tazobactam with or without aminoglycosides), norepinephrine with or without dobutamine in case of hemodynamic shock, infusion of iv insulin for glucose level control, furosemide with hemofiltration in oliguria or anuria, stress ulcer prophylaxis with omeprazole, and thrombosis prophylaxis with enoxaparine or fraxiparine were administered. Morphine and propofol were used for analgesia and sedation during mechanical ventilation at appropriate doses. None of the patients received drotrecogin alfa.

### Methods

All the experiments described here were performed in accordance with the regulations issued by the Ethics Committee of the HUCA.

### Plasma MMPs and TIMPs

Ten ml of blood were drawn by venipuncture in EDTA-containing tubes within the first 24 hours of ICU admission in all the individuals, and in a subgroup of 14 sepsis patients and 18 controls samples were also obtained at days 1, 3 and 7 of ICU stay. Tubes were centrifuged for 5 minutes at 1,800 g and serum was removed and frozen at −80°C until use. Plasma levels of MMPs-1, -2, -3, -8, - 9, -10 and -13 and TIMP -1, -2, and -4 were measured using the Quantibody^TM^ Human MMP Array 1 from Raybiotech (Raybiotech, Parkway Lane, Norcross, GA, USA) according to the manufacturer's instructions^26^.

### MMPs SNPs genotyping

DNA was obtained from peripheral blood cells and stored at −20°C before use. The following SNPs of MMPs were genotyped by PCR: *MMP-1 (−1607 1G/2G*, rs 11292517), *MMP-3 (−1612 5A/6A*, rs35068180), MMP-8 (−799 C/T, rs 11225395), *MMP-9 (−1562 C/T*, rs 34016235), and *MMP-13 (−77 A/G*, rs 2252070). Oligonucleotide primer sequences, PCR conditions and restriction enzymes used for genotyping and sequencing the different matrix metalloproteases (*MMPs*) polymorphisms studied are described elsewhere^16^,^22^,^24^,^26^.

### Other laboratory analysis

A complete hemogram, coagulation, general biochemistry, and C-reactive protein (CRP) levels were obtained from patients and controls at day 1 of ICU admission.

### Statistics

Results are expressed as median and inter-quartile range (IQR), mean and range or proportions as appropriate. Spearman's rho was used to assess the correlations among continuous variables. The Pearson χ^2^ test was used to compare allele and genotype frequencies and the clinical characteristics between the groups. Fisher' exact test and the Yates correction were used when indicated. Odds ratios (OR's) and their 95% confidence intervals (CI) were also calculated. Variables with a normal distribution were compared by the Student t test, while those without a normal distribution were compared by the Mann-Whitney test. A repeated measures general linear model procedure was carried out to evaluate the course of MMPs and TIMPs over time. Receiver operating characteristic (ROC) curves were used to evaluate the diagnostic accuracy of selected variables. A multivariate stepwise logistic regression analysis was also done to assess the factors independently associated with sepsis and death. The statistical analysis was performed with the SPSS Statistical Software (version 15.0, Chicago, USA) and GraphPad Prism Software (version 5.0, San Diego, CA, USA). All the reported p values are two-sided. A p-value < 0.05 was considered as statistically significant.

## Results

### Patients clinical and laboratory characteristics and outcome

Septic and uninfected ICU patients were matched in age and sex. There were no differences regarding the APACHE II score. A total of 21 individuals died (11.6%). Although more uninfected controls died there were not statistically significant differences compared to the septic patients. As expected, the number of organ failures has significantly higher in septic patients. Positive blood cultures were observed in 75 (83.3%) of septic patients, 40 (53.3%) of them grew Gram negative bacteria, mostly *Escherichia coli* (18, 24%) and 35 (46.7%) grew Gram positive bacteria, mostly *Streptococcus pneumoniae* (16, 21.3%). The main sources of infection were urinary, intra-abdominal and respiratory. The uninfected control group was composed of ICU patients admitted mainly because of politrauma and neurological problems.

Table 1 shows the demographic and clinical characteristics of both groups, and Table 2 the laboratory results. We observed significantly higher levels of neutrophils, CRP and plasma MMP-3, -8, -10, and TIMP-2 and lower concentrations of MMP-9 in septic patients compared to uninfected controls at day 1 of ICU admission. MMP-8 correlated positively (r = 0.39, p < 0.0001) and MMP-9 negatively (r = −0.29, p = 0.003) with CRP levels. The remaining MMPs and TIMPs did not show any significant correlation with CRP (p = 0.07 to p = 0.9). Plasma levels of MMP-2 and MMP-13 were significantly increased in septic survivors compared to non-survivors (Table 3).

### MMPs SNPs

**Figure 1:**
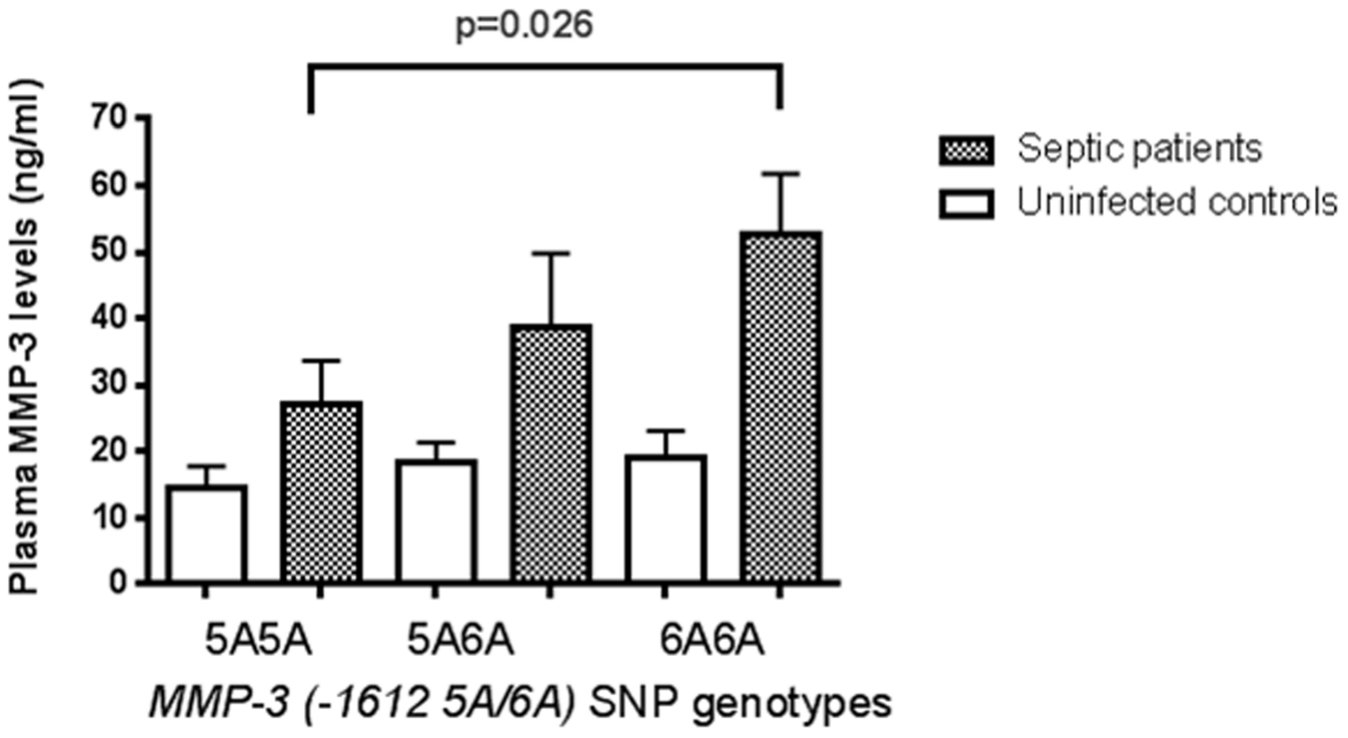
Plasma levels of MMP-3 at ICU admission in septic and uninfected control patients carriers of the different genotypes of the *MMP-3 (-1612 5A/6A)* SNP. Bars represent median and IQR values in ng/mL.

Table 4 shows the genotypic and allelic frequencies in septic and control patients. Of all the *MMPs* SNPs studied we only found that the *AA* genotype of the *MMP-13 (−77 A/G)* SNP was significantly more frequent and the *1G2G* genotype of the *MMP-1(−1607 1G/2G)* less frequent in septic patients compared to uninfected ICU controls. The *A* allele of the *MMP-13* SNP was also significantly more frequent in septic patients compared to uninfected controls. No differences in plasma MMPs levels in carriers of the different genotypes of the *MMP-13 (−77 A/G)* and of the *MMP-1(−1607 1G/2G)* SNPs were observed (data not shown). Septic carriers of the *MMP-3(−1612 5A/6A)* genotype had significantly higher plasma levels of MMP-3 compared to those with the *5A5A* genotype and to uninfected controls (Figure 1).

Due to the relatively small number of non-survivors in the sepsis group (7 patients) it is difficult to draw definitive conclusions regarding the *MMPs* SNPs studied and mortality. No associations with the APACHE II score or the number of organ failures were observed for any of the *MMPs* SNPs studied.

### Logistic regression analysis

Variables with a p value < 0.1 in the univariate comparisons were used for the logistic regression analysis. Sepsis was independently associated with the following variables at the first day of ICU admission: higher MMP-8 concentrations (OR [95%CI] 1.107 [1.058–1.158], p < 0.0001), fibrinogen levels (1.010 [1.005–1.015, p = 0.0001), neutrophils counts (1.116 [1.014–1.228], p = 0.02), and number of organ failures (2.174 [1.117–4.229], p = 0.02), and lower concentrations of MMP-9 (0.995 [0.992–0.998], p = 0.0001).

Survival in septic patients was independently associated with lower plasma levels of MMP-2 (OR [95%CI] 0.456 [0.252–0.824], p = 0.009) and higher prothrombin activity (1.107 [1.018–1.204], p = 0.018) in the multivariate regression analysis.

### ROC curves

The area under the ROC curve for MMP-8 in the diagnosis of sepsis was 0.87 (95% CI 0.82–0.92), p < 0.0001. The most discriminant value was 30.5 ng/mL (sensitivity 80.0%, specificity 84.6%). The area for MMP-9 in the detection of non-septic state was 0.73 (95% CI 0.65–0.80), p < 0.0001, being the most discriminant cut-off level 567.1 ng/mL (sensitivity 68.1%, specificity 70.0%). Regarding CRP, the area under the ROC curve in the diagnosis of sepsis was 0.98 (95% CI 0.94–0.998), p < 0.0001, better than those of the MMPs and TIMPs. The most discriminant value of CRP was 8.0 ng/mL (sensitivity 89.0%, specificity 92.3%).

### Plasma MMPs and TIMPs time course

**Figure 2:**
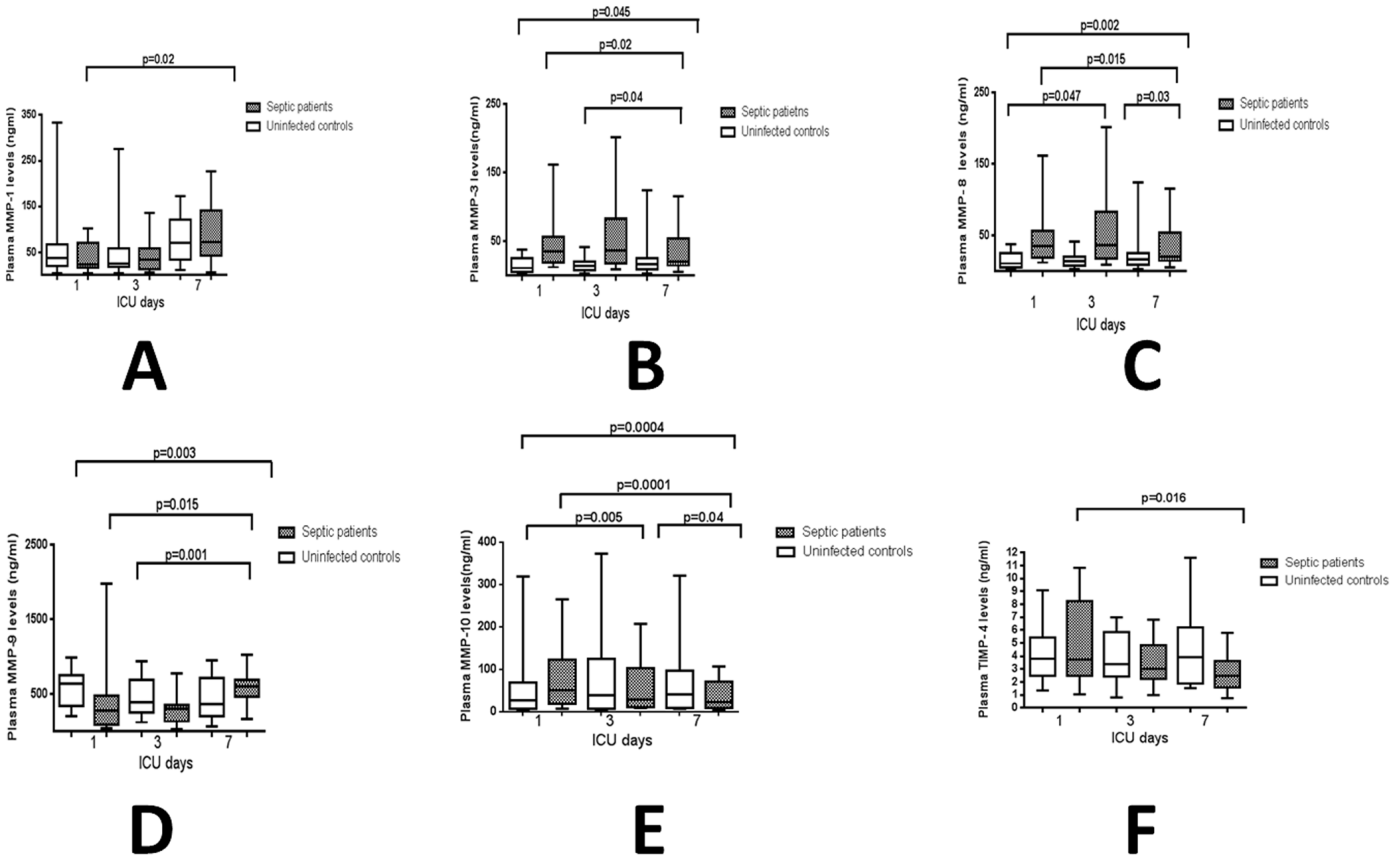
Time course plasma levels of MMP-1 (A), -3 (B), -8 (C), -9 (D), -10 (E) and TIMP-4 (F) at days 1, 3 and 7 of ICU admission. Boxes and whiskers represent mean and range values in ng/mL.

The course of MMPs y TIMPs over time were evaluated in a subgroup of 14 sepsis and 18 uninfected control patients, who underwent measurements at day 1, 3 and 7 of ICU admission. Considering the three determinations of each septic patient altogether, we found progressively decreasing levels over time for MMP-3 (p = 0.02), MMP-8 (p = 0.015), MMP-10 (p = 0.0001) and TIMP-4 (0.016), and progressively increasing concentrations for MMP-1 (p = 0.02) and MMP-9 (p = 0.01). On the contrary, there were no statistically significant differences over time for any MMP or TIMP in the control group (Fig. 2, A–F).

The slopes of the MMPs and TIMPs curves over time were significantly different between septic and uninfected control patients only for MMP-3 (overall p = 0.045; days 1–3 p = 0.6; days 3–7 p = 0.04), MMP-8 (overall p = 0.002; days 1–3 p = 0.047; days 3–7 p = 0.03), MMP-9 (overall p = 0.003; days 1–3 p = 0.5; days 3–7 p = 0.001) and MMP-10 (overall p = 0.0004; days 1–3 p = 0.005; days 3–7 p = 0.04).

## Discussion

We report here for the first time an association between the *MMP-13 (−77 A/G)* and the *MMP-1 (−1607 1G/2G)* SNPs and sepsis. The *MMP-13 SNP* association with sepsis we report might not be surprising considering the central role of MMP-13 in the MMPs activation cascade and the involvement of MMPs in inflammation and response to endotoxin^7^. However the mechanism by which carriers of the *AA* genotype and the *A* allele of this *MMP-13* SNP develop more easily sepsis is not well understood. We could not find an association between changes of plasma MMP-13 levels and the different genotypes of the *MMP-13* SNP. However, the expression of MMP-13 might be increased in collagen and other human stromal cells in carriers of the *MMP-13* SNP, a fact that invites further research and that we could not analyze because tissue biopsies were not obtained in our study. On the other hand increased expression of MMP-13 due to the *MMP-13* SNP might contribute to changes in the expression and plasma levels of other MMPs intimately bound to MMP-13 in the MMPs activation cascade such as MMP-2 (increased in plasma of non-surviving septic patients in our study) and MMP-9 (decreased in plasma of septic patients in our study). The association of the *MMP-1 (-1607 1G/2G)* SNP and sepsis might be due to linkage disequilibrium with the *MMP-13* SNP because the *MMP-13* and *MMP-1* genes are both located in the same cluster of the chromosome 11q22-23 along with the *MMP*-3, *MMP*-7, *MMP*-8, *MMP-10*, *MMP*-12, and *MMP*-20 genes. A common evolutionary ancestry for the M*MPs* genes located on chromosome 11q has been suggested^7^. Borghese *et al* have confirmed the linkage disequilibrium between the *MMP-13* and *MMP-1* SNPs in a large multi-ethnic genetic study. In addition they reported allelic frequencies of the *MMP-1*, *MMP-3* and *MMP-13* SNPs in healthy French Caucasians women similar to those observed in the ICU uninfected controls in our study^24^.

The association of *MMPs* SNPs and infection was been rarely explored so far. We have previously reported an association of the *MMP-1* *(-1607 1G/2G*, rs 11292517) SNP with bacterial osteomyelitis due to an increased bone expression and plasma levels of MMP-1 in carriers of *2G2G* genotype of the *MMP-1* SNP^26^. Pan *et al* also reported an association of chronic periodontitis with the carriage of the *TT* genotype of the *MMP-9 (-1562 C/T*, rs 34016235) SNP among Chinese individuals^25^.

The *6A6A* genotype of the *MMP-3(-1612 5A/6A)* SNP has been previously associated with a lower gene expression of the *MMP-3* gen and lower MMP-3 plasma levels compared to those carriers of the *5A5A* genotype^16^,^17^,^18^,^19^. However other studies have reported that carriers of the *6A6A* genotype of this *MMP-3* SNP had higher plasma MMP-3 levels^20^,^21^,^22^,^23^,^24^,^25^,^26^,^27^,^28^,^29^,^30^,^31^,^32^,^33^,^34^. Our results were similar to the latter studies although only in septic patients but not in the uninfected controls, which is intriguing and needs further exploration. The *MMP-3* SNP has been associated with different forms of cardiovascular disease, including severe coronary disease and myocardial infarction but not with sepsis or infection^16^,^33^,^34^.

We also observed, as other authors did, increased plasma levels of most of the MMPs, except MMP-9, and TIMP-2 in septic patients but not in the uninfected controls. CRP plasma levels correlated positively with those of MMP-8 and negatively with those of MMP-9. Plasma MMP-9 levels were lower in septic patients compared to those of the controls, a fact to consider because decreased plasma MMP-9 levels were associated with sepsis in the multivariate logistic regression in our study. The implication of gelatinases (MMP-2 and MMP-9), both secreted by the circulating neutrophils, in sepsis pathophysiology is well documented^8^,^10^,^11^,^12^,^13^,^14^,^15^. We bring forward now the potential role of collagenases (MMP-13 and MMP-1) and perhaps stromelysins (MMP-3) in sepsis, an interesting finding because these MMPs are neither expressed in neutrophils nor released from neutrophils granules after endotoxin challenge as MMP-8 and MMP-9 do^5^. The association of MMP-1 with sepsis has been recently reported. Tressel *et al* observed that MMP-1 appeared just a few hours after sepsis in human septic patients and in a murine model. In addition a MMP-1 inhibitor blocked experimentally-induced sepsis in the same animals^35^. MMP-1 is expressed by unstressed endothelial cells and secreted by these cells in sepsis. This fact leads to protease-activated receptor 1 (PAR1) activation, vascular permeability, clotting abnormalities and production of cytokines and prostaglandins^36^.

Regarding the MMPs and TIMPs time course it is important to keep in mind that MMP-1, and MMP-9 plasma levels in septic patients peaked at day 7 of ICU stay from admission levels in our study. On the contrary MMP-3, MMP-8, MMP-10 and TIMP-4 had their nadir serum levels at day 7 of ICU stay. This might be one of the reasons of the disparity in plasma MMPs levels data in our study compared to previous reports in which blood was mostly drawn only within the day 1 or at random during the ICU stay. We could not find differences between TIMP-1 plasma levels of ICU septic and uninfected patients or among non-surviving and surviving septic patients. TIMP-1 has been proposed as a useful laboratory marker to predict the clinical outcome of septic patients^10^,^11^. Furthermore, carriers of the *T* allele in the *TIMP-1 (372 T/C)* SNP (rs4898) showed higher serum TIMP-1 levels and lower survival rate among septic patients in one study^27^. The low death rate among septic patients in our cohort (7.8%) has limited the statistical significance of TIMP-1 and the rest of data analyzed. The low mortality rate in this septic cohort of ICU patients with a mean of 3 organ failures might be due to the very restrictive inclusion criteria we used in our study excluding patients with cancer, HIV infection, transplantation or other causes of immunodepression, patients normally included in previous ICU sepsis series. This restrictive inclusion criteria has also limited greatly the number of septic patients enrolled and expanded the recruitment window of the study to more than 3 years.

We can summarize our findings on MMPs in sepsis pathophysiology as follows. Bacterial endotoxin stimulates the release of cytokines that contribute to the expression and release of MMPs from endothelial cells (MMP-1) or from neutrophils (MMP-8 and MMP-9). In addition MMPs activate cytokines precursors in a self-perpetuating vicious circle. MMP-13 has a leading role in the MMPs-activation process and a *MMP-13* SNP influencing MMP-13 expression might contribute to sepsis enhancement as we found^7^. Among the MMPs showing earlier increased plasma levels in sepsis were MMP-3, -8, and -10 as we observed while others like MMP-1 and -9 increased later. High MMP-8 and low MMP-9, which correlated with plasma CRP levels, were associated with sepsis, in addition to high fibrinogen levels and neutrophils counts. Some of the MMPs laterly increased in plasma, such as MMP-1, lead to sepsis-associated clotting abnormalities. Other MMPs through membranes basement ECM collagen cleavage help neutrophils crossing from blood and lymph vessels to the infection locus. Finally some MMPs, including MMP-1, contribute to tissue repair once the infection has subsided^5^.Therefore a *MMP-1* SNP that influence MMP-1 expression might play a dual effect in both sepsis enhancement and in inflammation-damaged tissue repair.

Some papers based on the role of MMPs and TIMPs in sepsis have been published in the last 15 years^8^,^9^,^10^,^11^,^12^,^13^,^14^,^15^. The main limitations of these studies are: 1. small size; 2. enrollment of healthy controls instead of ICU uninfected patients; and 3. the lack of exclusion criteria for patients with underlying immunodeficiencies^37^. Our cohort, made of 90 septic patients was the only one enrolled from one single hospital. In addition we included uninfected 91 ICU patients as controls instead of healthy individuals, something rarely done and we excluded patients with underlying immunodeficiencies, a fact not previously considered. The main limitations of our study in addition to the relatively small sample size and the low septic patients mortality is our only Caucasian population. More research in multi-ethnic larger populations of septic patients is needed to confirm our findings.

In conclusion, we report for the first time an association between the *MMP-13* and the *MMP-1* SNPs and sepsis, as well as an independent association of plasma MMP-8 and MMP-9 levels with sepsis. The role of MMPs and their SNPs in sepsis development needs further research.

## Author Contributions

G.M., J.A.C. and F.T. recruited the ICU patients and contributed to design the study. A.M. and V.A.^5^ did the MMPs genotyping. E.V.G. measured the MMPs plasma levels and contributed to design the study. J.C. did the statistical analysis of the data and edited the manuscript. V.A.^1^ designed the study, wrote the main manuscript text and prepared the figures 1 and 2.

## Figures and Tables

**Table 1:** Overall demographic and clinical characteristics of septic and uninfected ICU patients

	Septic (n = 90)	Uninfected Controls (n = 91)	p-value
Age	58.5 (45.0–73.0)	64.0 (50-0–71.0)	0.9
Gender (male/female)	50/40	61/30	0.1
APACHE II	17.0 (12.0–23.0)	16.0 (9.0–22.0)	0.2
Mortality (%)	7 (7.8)	14 (15.4)	0.1
Number of organ failures Source of infection	3.0 (2.0–4.0)	2.0 (1.0–2.0)	<0.0001
Genito-urinary (%)	26 (28.9)		
Intra-abdominal (%)	22 (24.4)		
Lung (%)	16 (17.8)	-	
CNS (%)	12 (13.3)	-	
Skin-soft tissue (%)	8 (8.9)	-	
Endocarditis (%)	5 (5.6)	-	
Other location (%)	1 (1.1)	-	
Non-septic diagnosis			
Polytrauma (%)	-	40 (44.0)	
Cerebro-vascular attack (%)	-	46 (50.5)	
Other diagnosis (%)	-	5 (5.5)	

Results are expressed as median (IQR).

**Table 2:** Laboratory results of septic and uninfected control patients at day 1 of ICU admission

	Septic (median, IQR)	Uninfected (median, IQR)	p-value
Number	90	91	
Neutrophils (x10^3^/mL)	12.8 (7.5–19.5)	9.6 (5.8–14.5)	0.002
CRP (mg/mL)	26.0 (17.6–36.2)	1.0 (0.3–3.1)	<0.0001
Fibrinogen (mg/dL)	692.0 (576.5–847.8)	433 (322.8–556.39)	<0.0001
Prothrombin complex factors rate (%)	59.5 (49–74)	83 (72–92)	<0.0001
Activated partial tromboplastine time (seconds)	28.9 (17–8-35.4)	26.1 (21.38–29)	0.02
MMPs/TIMPs (ng/mL)			
MMP-1	25.6 (15.5–57.6)	27.8 (14.5–56.1)	0.5
MMP-2	1.0 (0.5–1.0)	0.6 (0.4–1.1)	0.1
MMP-3	19.8 (12.1–47.2)	12.4 (6.6–23.9)	<0.0001
MMP-8	48.0 (33.7–73.0)	16.5 (10.1–35.7)	<0.0001
MMP-9	394.3 (198.8–618.7)	592.4 (376.6–766.9)	<0.0001
MMP-10	50.9 (27.8–160.4)	23.2 (10.4–47.7)	<0.0001
MMP-13	0.1 (0.07–0.3)	0.08 (0.04–0.2)	0.2
TIMP-1	253.1 (230.6–304.6)	259.3 (218.0–303.6)	0.9
TIMP-2	101.7 (70.3–124.7)	87.2 (58.8–119.1)	0.045
TIMP-4	3.7 (2.1–6.9)	2.8 (1.8–5.5)	0.07

**Table 3:** Laboratory characteristics of surviving and non-surviving ICU septic patients

	Non-survivors (median, IQR)	Survivors (median, IQR)	p-value
Number	7 (7.8)	83 (92.2)	
Neutrophils (x10^3^/mL)	7.5 (43.1–16.4)	13.5 (7.7–19.6)	0.1
CRP (mg/mL)	25.1 (9.3–28.6)	26.6 (17.9–37.1)	0.5
Fibrinogen (mg/dL)	506.5 (267.3–738.0)	703 (585.5–870)	0.026
Prothrombin complex factors rate (%)	36 (26–58.8)	60 (50.3–74.8)	0.004
Activated partial tromboplastine time (seconds)	29.0 (21.1–37.4)	28.8(17.7–35.3)	0.9
MMPs/TIMPs (ng/mL)			
MMP-1	54.7 (34.1–85.1)	24.7 (14.8–50.9)	0.051
MMP-2	5.4 (1.3–6.8)	0.8 (0.5–1.8)	0.001
MMP-3	45.2 (29.7–65.4)	19.0 (11.9–45.6)	0.1
MMP-8	55.2 (30.6–96.7)	47.7 (33.9–71.3)	0.4
MMP-9	164.0 (121.9–484.0)	399.1 (212.1–629.6)	0.1
MMP-10	96.6 (46.1–172.1)	49.5 (26.9–156.3)	0.3
MMP-13	0.4 (0.1–0.6)	0.1 (0.07–0.2)	0.04
TIMP-1	235.4 (188.2–323.7)	256.6 (230.7–304.1)	0.4
TIMP-2	106.9 (70.5–180.1)	99.8 (69.9–124.1)	0.6
TIMP-4	4.0 (3.1–25.6)	3.6 (2.0–6.6)	0.2

**Table 4:** Genotypic and allelic frequencies of different *MMPs* SNPs in ICU septic patients and uninfected controls

	Genotypic frequencies				Allelic frequencies			
SNP	Genotype	Septic n (%)	Controls n (%)	p value	Allele	Septic n (%)	Controls n (%)	p value
*MMP-13 (-77A/G)*	*AA*	54 (60.0)^a^	39 (42.9)	0.03	*A*	144 (80.0)^b^	129 (70.9)	0.044
	*AG*	36 (40.0)	51 (56.0)		*G*	36 (20.0)	53 (29.1)	
	*GG*	0 (0.0)	1 (1.1)					
	n	90	91					
								
*MMP-1* (-1607 1G/2G)	*1G1G*	31 (34.8)	25 (27.5)	0.013	*1G*	88 (49.4)	94 (51.6)	0.7
	*1G2G*	26 (29.2)^c^	44 (48.3)		*2G*	90 (50.6)	88 (48.4)	
	*2G2G*	32 (36.0)	22 (24.2)					
	n	89	91					
								
*MMP-3 (-1612 5A/6A)*	*5A5A*	29 (32.2)	26 (28.6)	0.3	*5A*	94 (52.2)	99 (54.4)	0.7
	*5A6A*	36 (40.0)	47 (51.6)		*6A*	86 (47.8)	83 (45.6)	
	*6A6A*	25 (27.8)	18 (19.8)					
	n	90	91					
								
*MMP-8 (-799C/T)*	*CC*	20 (23.3)	23 (25.8)	0.5	*C*	84 (48.8)	96 (53.9)	0.3
	*CT*	44 (51.1)	50 (56.2)		*T*	88 (51.2)	82 (46.1)	
	*TT*	22 (25.6)	16 (18.0)					
	n	86	89					
								
*MMP-9 (-1562 C/T)*	*CC*	72 (80.0)	69 (76.7)	0.6	*C*	162 (90.0)	159 (88.3)	0.6
	*CT*	18 (20.0)	21 (23.3)		*T*	18 (10.0)	21 (11.7)	
	*TT*	0 (0.0)	0 (0.0)					
	n	90	91					

^a^χ^2^ = 4.66, OR (95%CI) = 2.0 (1.05–3.78) comparing the *AA* genotypic frequencies between septic and control patients.

^b^χ^2^ = 3.58, OR (95%CI) = 1.64 (0.98–2.75) comparing the *A* allelic frequencies between septic and control patients.

^c^χ^2^ = 6.15, OR (95%CI) = 0.44 (0.23–0.85) comparing the *1G2G* genotypic frequencies between septic and control patients.
